# Draft Genome Sequences of 15 Isolates of *Listeria monocytogenes* Serotype 1/2a, Subgroup ST204

**DOI:** 10.1128/genomeA.00935-16

**Published:** 2016-09-08

**Authors:** Theodore R. Allnutt, Mark I. Bradbury, Séamus Fanning, P. Scott Chandry, Edward M. Fox

**Affiliations:** a CSIRO Agriculture and Food, Werribee, Victoria, Australia; b School of Medicine, Deakin University, Waurn Ponds, Victoria, Australia; c CSIRO Agriculture and Food, North Ryde, Sydney, Australia; d UCD Centre for Food Safety, School of Public Health, Physiotherapy & Population Science, University College Dublin, Dublin, Ireland

## Abstract

*Listeria monocytogenes* sequence type 204 (ST204) strains have been isolated from a range of food, environmental, and clinical sources in Australia. This study describes the draft genome sequences of 15 isolates collected from meat and dairy associated sources.

## GENOME ANNOUNCEMENT

*Listeria monocytogenes* is a Gram-positive bacterial pathogen and the causative agent of listeriosis. Human infection is frequently associated with the consumption of contaminated foods (1). A total of 15 serotype 1/2a isolates identified as sequence type (ST) 204 were collected from food processing environments: 14 from Australia and one from the Republic of Ireland.

DNA from each isolate was purified using the DNeasy blood and tissue kit (Qiagen, the Netherlands), as per manufacturer's instructions. Library preparation using the Nextera XT library prep kit and 250-bp paired-end sequencing were performed using the Illumina MiSeq platform (Illumina, San Diego, CA). Raw reads were preprocessed to remove adapter sequences and low-quality reads using Trimmomatic (v0.22) software (2). *De novo* assembly was performed using SPAdes genome assembler (v1.0) (3).

Assemblies ranging between 21 and 89 contigs were generated per genome with total sizes ranging from 2.94 Mbp to 3.11 Mbp (mean = 3.01 Mbp). G+C content ranged from 37.7% to 38.0%. Draft genomes were annotated using the online tool, RAST (4). The number of coding sequences (CDS) per genome ranged from 2,886 to 3,096, accounting for 89.6% to 90.6% of the genome. Plasmid contigs were identified using a local protein-protein BLAST search (BLAST 2.2.31+, http://www.ncbi.nlm.nih.gov/news/06-16-2015-blast-plus-update/) against a database of known *L. monocytogenes* plasmid proteins, retrieved from the NCBI protein search, using the term: “‘*Listeria monocytogenes*’[Organism] AND plasmid[All Fields]” (5,925 proteins). The *N*_50_ values for isolates are listed in Table 1.

**TABLE 1:** Genome assembly details and statistics

Isolate	NCBI BioSample no.	GenBank accession no.	Genome size (bp)	No. of contigs	*N*_50_ (bp)
2882	SAMN04932619	LXQP00000000	3,037,029	50	435,670
2919	SAMN04932620	LXQQ00000000	3,113,342	44	373,241
2937	SAMN04932621	LXQR00000000	2,949,465	49	435,670
2939	SAMN04932622	LXQS00000000	2,948,045	46	289,670
2945	SAMN04932623	LXQT00000000	3,084,502	81	405,327
2964	SAMN04932624	LXQU00000000	3,047,204	59	373,213
2973	SAMN04932625	LXQV00000000	3,050,778	79	431,256
2977	SAMN04932626	LXQW00000000	2,991,193	87	92,345
2978	SAMN04932627	LXQX00000000	3,029,546	56	342,713
2981	SAMN04932628	LXQY00000000	3,082,357	64	291,396
3002	SAMN04932629	LXQZ00000000	2,983,094	46	435,692
Lm15-001	SAMN04932630	LXRA00000000	2,973,454	35	291,742
Lm15-011	SAMN04932631	LXRB00000000	3,014,557	89	115,048
Lm15-027	SAMN04932632	LXRC00000000	2,954,279	42	217,080
UCDL175	SAMN04932633	LXRD00000000	3,020,537	21	435,670

Of the 15 isolates, 13 contained contigs harboring plasmid elements, with the remaining two containing no plasmid. Plasmids contained genes encoding a variety of functions including heavy metal resistance and stress response. Four prophage insert regions were identified among isolates using the online tool PHAST (5), located within the monocin locus, the *comK* gene, upstream of an RNA methyltransferase gene (*lmo1703* homolog) and downstream of tRNA-Arg-TCT. Two transposon loci were identified, including an insertion in the y*fbR* gene and another in an internalin-like protein coding gene (*lmo2026* homolog).

### Accession number(s).

This whole-genome shotgun project has been deposited at DDBJ/ENA/GenBank under the accession numbers listed in Table 1.

## References

[1] Scallan E, Hoekstra RM, Angulo FJ, Tauxe RV, Widdowson MA, Roy SL, Jones JL, Griffin PM 2011 Foodborne illness acquired in the United States—major pathogens. Emerg Infect Dis 17:7–15. doi:10.3201/eid1701.091101p1. 21192848 PMC3375761

[2] Bolger AM, Lohse M, Usadel B 2014 Trimmomatic: a flexible trimmer for Illumina sequence data. Bioinformatics 30:2114–2120. doi:10.1093/bioinformatics/btu170. 24695404 PMC4103590

[3] Bankevich A, Nurk S, Antipov D, Gurevich AA, Dvorkin M, Kulikov AS, Lesin VM, Nikolenko SI, Pham S, Prjibelski AD, Pyshkin AV, Sirotkin AV, Vyahhi N, Tesler G, Alekseyev MA, Pevzner PA 2012 SPAdes: A new genome assembly algorithm and its applications to single-cell sequencing. J Comput Biol 19:455–477. doi:10.1089/cmb.2012.0021. 22506599 PMC3342519

[4] Aziz RK, Bartels D, Best AA, DeJongh M, Disz T, Edwards RA, Formsma K, Gerdes S, Glass EM, Kubal M, Meyer F, Olsen GJ, Olson R, Osterman AL, Overbeek RA, McNeil LK, Paarmann D, Paczian T, Parrello B, Pusch GD 2008 The RAST server: Rapid Annotations using Subsystems Technology. BMC Genomics 9:75. doi:10.1186/1471-2164-9-75. 18261238 PMC2265698

[5] Zhou Y, Liang Y, Lynch KH, Dennis JJ, Wishart DS 2011 PHAST: A fast phage search tool. Nucleic Acids Res 39:W347–W352. doi:10.1093/nar/gkr485. 21672955 PMC3125810

